# CHILDREN’S HEALTH: Coal Fire Emissions Curb Children’s Growth

**DOI:** 10.1289/ehp.119-a246a

**Published:** 2011-06

**Authors:** Adrian Burton

**Affiliations:** **Adrian Burton** is a biologist living in Spain who also writes regularly for *The Lancet Oncology*, *The Lancet Neurology*, and *Frontiers in Ecology* and the Environment

Putting more coal on the fire may help to keep off the chill, but it may not help your baby grow. New research found that the 3-year-old children of Czech families who reported using coal for indoor heating were shorter than those whose families used cleaner fuels.^1^

Similar to cigarette smoke, the smoke of burning coal contains fine particles, carbon monoxide, benzene, polycyclic aromatic hydrocarbons (PAHs), sulfur dioxide, arsenic, and other toxicants.^2^ “PAHs and particulate matter are associated with reduced intrauterine growth,” explains Irva Hertz-Picciotto, a professor of public health sciences at the University of California, Davis, and senior author of the study. “Air pollution has been linked to smaller length and head circumferences at birth, and there is evidence that secondhand [tobacco] smoke can affect the stature of children.” Putting the pieces together, she says she and her colleagues became interested in whether indoor coal burning—a common exposure scenario in many countries—might affect children’s early postnatal growth.

To investigate this potential link, the researchers examined the height at 36 months of 1,105 Czech children whose mothers had been recruited into the Teplice Pregnancy Outcome Study launched by the Czech government with assistance from the U.S. Environmental Protection Agency. These families were then followed longitudinally in the Children’s Health and Air Pollution Study; information was obtained from pediatricians about the children’s health and growth at birth and at 36 months, and the children’s mothers responded to questionnaires to determine how families heated their homes, their children’s exposure to secondhand tobacco smoke, and other lifestyle variables.

The researchers used the medical history data to determine height-for-age-and-sex *z* scores for the children in homes that did and did not use coal for indoor heating. “These scores reflect the difference between height-for-age-and-sex of a child compared with a reference population, with the units being standard deviations,” Hertz-Picciotto explains. “The growth of a child with a negative score is below the mean for the reference population, and the lower the score, the poorer the growth.”

Indoor coal combustion was used to heat 10.2% of the children’s homes; of these, 77.6% used coal exclusively. The mean height-for-age-and-sex at 36 months for the children in these homes was significantly lower than that of children from non-coal-burning homes. In regression modeling, adjusting for confounders such as birth weight for gestational age and sex, maternal height, and maternal ethnicity, height-for-age-and-sex was found to be significantly associated with indoor coal combustion.

Translating the *z* scores into absolute height differences, 3-year-old boys from indoor coal-burning homes were a mean 1.34 cm shorter, while the girls were a mean 1.3 cm shorter, says first author Rakesh Ghosh, a postdoctoral scholar at UC Davis. “The results showed the effect to be compounded if children were [also] exposed to secondhand cigarette smoke,” Ghosh says.

The link between respiratory disease in children and the indoor burning of solid fuels (including coal, wood, and dung) has long been known in the developing world, where homes commonly have open hearths and no chimney. But this is the first time growth reduction has been associated with coal burning in the ventilated indoor hearths and furnaces of a developed country. Even with ventilated coal heaters, Hertz-Picciotto says there are two ways children can be exposed: first, when the coal is added, smoke and ash likely enter the room; and second, some fraction of the particles vented outdoors likely land nearby and circulate in areas near the house.

The World Health Organization describes the emissions from solid fuels as the “killer in the kitchen,” claiming them responsible for 1.5 million respiratory illness–related deaths every year, mostly in Southeast Asia and sub-Saharan Africa and predominantly affecting women and children.^3^ Some research has also linked the use of such fuels to poorer growth in young children in developing countries,^4^,^5^ whereas other work has associated indoor coal use with increased lower respiratory tract illnesses.^6^

“The results provide an important public health warning for countries where coal is still burned indoors,” says study co-supervisor Radim Šrám of the Institute of Experimental Medicine AS CR, Prague, Czech Republic. “Moving to cleaner heating systems should be a priority, yet indoor coal use in some countries, such as China, is increasing.”

“It is difficult to disentangle the many influences on child development,” points out Martin McKee, a professor of European public health at the London School of Hygiene & Tropical Medicine, who was not involved with the study. “Many of them cluster together, so that families that are disadvantaged in one way may be exposed to many different hazards. As the authors note, they were unable to assess the extent to which children were exposed to coal smoke, [but nonetheless, this study] raises some important questions.”

## Figures and Tables

**Figure:**
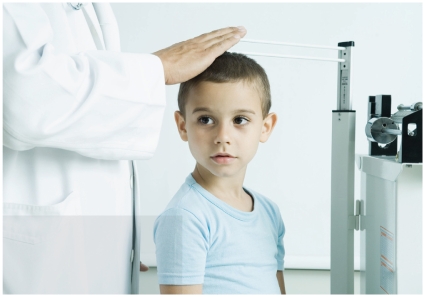
Multiple compounds in coal smoke are associated with growth deficits in children.
